# Epigenetics of cutaneous T-cell lymphoma: biomarkers and therapeutic potentials

**DOI:** 10.20892/j.issn.2095-3941.2020.0216

**Published:** 2021-02-15

**Authors:** Pan Lai, Yang Wang

**Affiliations:** 1 Department of Dermatology and Venereology, Peking University First Hospital, Beijing Key Laboratory of Molecular Diagnosis on Dermatoses, National Clinical Research Center for Skin and Immune Diseases, Beijing 100034, China

**Keywords:** Epigenetics, cutaneous T-cell lymphoma, DNA methylation, histone modification, microRNA, chromatin-remodeling complex

## Abstract

Cutaneous T-cell lymphomas (CTCLs) are a heterogeneous group of skin-homing non-Hodgkin lymphomas. There are limited options for effective treatment of patients with advanced-stage CTCL, leading to a poor survival rate. Epigenetics plays a pivotal role in regulating gene expression without altering the DNA sequence. Epigenetic alterations are involved in virtually all key cancer-associated pathways and are fundamental to the genesis of cancer. In recent years, the epigenetic hallmarks of CTCL have been gradually elucidated and their potential values in the diagnosis, prognosis, and therapeutic intervention have been clarified. In this review, we summarize the current knowledge of the best-studied epigenetic modifications in CTCL, including DNA methylation, histone modifications, microRNAs, and chromatin remodelers. These epigenetic regulators are essential in the development of CTCL and provide new insights into the clinical treatments of this refractory disease.

## Introduction

Cutaneous T-cell lymphomas (CTCLs) are a heterogeneous group of lymphoproliferative disorders characterized by the infiltration of skin-homing malignant T lymphocytes^1^. The most common variants are mycosis fungoides (MF) and Sézary syndrome (SS), which account for more than 60% of all CTCLs^1^. MF generally manifests as a low level lymphoma with an indolent clinical course, presenting as erythematous patches and plaques. Some patients inevitably progress to advanced stages with skin tumor and extra-cutaneous dissemination of malignant T cells to lymph nodes, blood, and visceral organs^1^. SS is a leukemic variant of CTCL that features aggressive disease progression with systemic involvement and poor prognosis^1^. Although 71% of patients present with early stage disease, disease progression occurs in 34% and 26% of patients with MF and SS, respectively^2^. Cutaneous CD30+ lymphoproliferative disorder (CD30+LPD) is the second most common form of CTCL, comprising a spectrum of diseases that range from lymphomatoid papulosis (LyP) to primary cutaneous anaplastic large cell lymphoma (PCALCL), which are characterized by a recurrent course and favorable prognosis^3^.

The treatment of CTCL primarily depends on the stage of the disease and escalates in a stepwise manner. The management of early stage disease (stages IA-IIA) focuses on skin-directed therapy consisting of topical agents, ultraviolet phototherapy, and local radiotherapy^4,5^. Systemic biological agents, including interferons or retinoids, are needed in patients with more extensive infiltration^4,5^. Systemic chemotherapy is usually reserved for patients with advanced or refractory/recurrent (R/R) CTCL^6,7^. Although good responses are reported with both single agent and combination chemotherapy regimens, the overall outcomes are disappointing when compared with other lymphomas^4^. The most commonly reported regimen used in CTCL is CHOP (cyclophosphamide, doxorubicin, vincristine, and prednisone). Recent evidence-based studies reported that the overall survival rates remain unchanged for more intensive regimens and these regimens have little clinical benefit compared with less intensive regimens^4^. Antibody therapies (mogamulizumab, alemtuzumab, and brentuximab vedotin) with significant clinical benefits have been developed in recent years^4^. However, the treatments for advanced CTCLs are mostly palliative, not curative, except for allogeneic stem cell transplantation, but the optimal regimen and timing remain unclarified^4^. Therefore, the development of effective treatments for patients with advanced CTCL is urgently needed. The molecular pathogenesis of CTCL remains largely unknown. Even in the era of next-generation sequencing (NGS), only a few genetic defects with pathogenic significance have been identified in CTCL^8,9^. Recurrent cancer mutations that have been described, including mutations in *PLCG1*, are restricted to a small portion of CTCL patients^10^. The cytogenetic drivers in most CTCL patients therefore remain to be elucidated.

Epigenetics is defined as a stably heritable phenotype resulting from changes in the chromosome without alterations in the DNA sequence. Epigenetics plays a central role in the pathogenesis of various cancers, including CTCL^11,12^. Substantial evidence now supports the effects of the epigenome on every component of gene regulation, including DNA methylation, post-translational histone modifications, chromatin structure, and microRNAs (miRNAs). Furthermore, epigenetic changes regulate a wide variety of cellular processes, including cell survival, proliferation, differentiation, and apoptosis^12^. The classical hallmarks of human cancer can potentially be achieved purely through epigenome deregulation^11^. Preclinical studies have shown that epigenetic alterations are potentially reversible through pharmacological manipulation, and the list of available epigenetic modifiers is steadily growing^13^. Moreover, epigenetic markers can be exploited as clinically relevant disease biomarkers for diagnosis, prognostication, and prediction of treatment responses^12^.

Over the past two decades, studies on epigenetic changes have identified the missing link between lymphoma-specific gene expression patterns and the absence of genetic alterations in CTCL^14–17^. These findings have improved our understanding of CTCL pathophysiology and facilitated the discovery of new disease biomarkers and therapeutic targets. More importantly, not all cancers are equally susceptible to epigenetic therapies. It is now evident from both clinical and preclinical studies that hematopoietic malignancies, including CTCLs, are more vulnerable to epigenetic interventions than solid malignancies. For example, azacitidine, a DNA methyltransferase (DNMT) inhibitor, has been approved by the Food and Drug Administration (FDA) for the treatment of myelodysplastic syndromes^18^. Clinical responses to another DNMT inhibitor, decitabine, are encouraging in elderly patients with acute myeloid leukemia^19^. The histone deacetylase inhibitors (HDACis), vorinostat and romidepsin, as two epigenetic drugs (“epi-drugs”) approved by the FDA for R/R CTCL patients, provide evidence to support epigenetic intervention as a promising treatment for this disease^20,21^.

**Figure 1:**
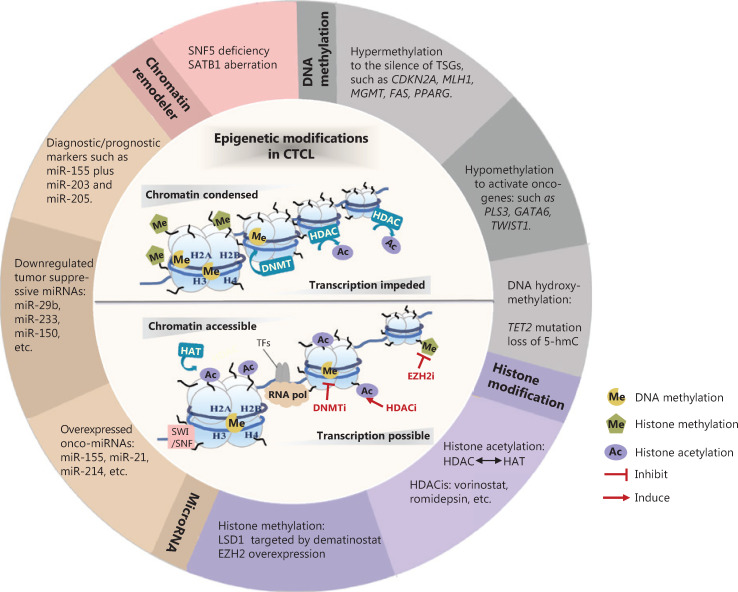
Epigenetic modifications in CTCL. Epigenetic alterations are implicated in the pathogenesis of CTCL, involving DNA methylation, histone modification, microRNA, and chromatin remodelers. These aberrant epigenetic modifiers demonstrate a broad role in altering chromatin accessibility status and regulating the transcriptional expression of a variety of tumor-related genes. Some “epi-drugs” exert promising antitumor effects in CTCL by targeting the key epigenetic enzymes. TSGs, tumor suppressor genes; 5-hmc, 5-hydroxymethylcytosine; HDAC, histone deacetylase; HAT, histone acetylase; HDACi, histone deacetylase inhibitor; LSD1, lysine-specific histone demethylase 1A; EZH2, enhancer of zeste homolog 2; onco-miRNAs, oncogenic microRNAs; miRNAs, microRNAs; SNF5, SWI/SNF chromatin-remodeling complex subunit SNF5; SATB1, Special AT-rich region binding protein 1; DNMT, DNA methyltransferase; SWI/SNF, switching defective/sucrose nonfermenting; TFs, transcription factors; RNA pol, RNA polymerase; DNMTi, DNA methyltransferase inhibitor; EZH2i, enhancer of zeste homolog 2 inhibitor.

Our aims in this review are to describe the main epigenetic alterations in CTCL, with an emphasis on their essential roles in the pathogenesis of CTCL, and to describe how these aberrations can potentially be utilized in clinical settings (**Figure 1**).

## DNA methylation

The earliest indications of an epigenetic link to cancer were derived from gene expression and DNA methylation studies^22–24^. DNA methylation is one of the most ubiquitous epigenetic modifications regulating gene expression^24–27^. The best-characterized DNA methylation process involves the addition of a methyl group (CH3) at the C5 position of the cytosine ring by DNMTs, yielding 5-methylcytosine (5-mC)^28^. DNMTs include DNMT1, which maintains preexisting methylation patterns, and DNMT3A and DNMT3B, which establish new sites of methylation^29,30^. Although global hypomethylation is commonly observed in malignant cells, the best-studied epigenetic alterations in cancer are the methylation changes that occur within CpG islands, which are present in 70% of all mammalian promoters^23,31^. CpG islands are CpG-rich sequences that are generally unmethylated in mammals and usually contain 200 to 2,000 nucleotides, of which > 50% are CpGs. Approximately 60% to 70% of gene promoters contain CpG islands^31–34^. Promoter methylation is the best-studied epigenetic mediator of oncogenic effects^35^. Using powerful techniques for the investigation of DNA methylation, such as sodium bisulfite conversion, CpG-island microarrays, and restriction landmark genomic scanning, an array of altered promoter methylation has been identified in CTCL. **Table 1** lists methylation alterations in genes with potential tumor suppressor or oncogene functions in CTCL and their potential roles in clinical settings.

**Table 1:** The aberrantly-methylated genes and their functions in cutaneous T-cell lymphomas

Methylation aberrancy	Function	Cellular effect	Gene	Methylation frequency*	Potential clinical value	References
Hyper-	Tumor suppressor	Cell cycle regulators	*CDKN1B*	73%		^37^
			*CDKN2B*	10%–36%		^14,172^
			*CDKN2A*	31%–36%		^14,37,172^
		Cell differentiation	*PPARG*	33%	Prognosis of MF	^37^
			*RARB*	27%		^37^
		Apoptosis signaling	*TMS1*	10%		^14^
			*FAS*	NA		^17,38^
			*TP73*	48%		^14^
		DNA repair	*MLH1*	16%–64%		^37,40^
			*MGMT*	33%–36%		^37,172^
		Chromosomal instability	*CHFR*	19%		^14^
		Insulin growth factor pathway	*IGF2*	57%		^37^
			*SOCS1*	19%		^37^
		Pluripotent regulation of stem cells	*NEUROG1*	37%		^37^
		Putative tumor suppres sors	*BCL7A*	48%		^14^
			*THBS4*	52%		^14^
			*PTPRG*	27%		^14^
			*CMTM2*	NA	Diagnosis of SS	^14^
			*SHP-1*	NA		^41,42^
			*SAMHD1*	NA		^173^
			*CD26*	NA	Prognosis	^39^
Hypo-	Oncogene	Actin-bundling protein	*PLS3*	60%SS 38%MF		^49,50^
		Candidate oncogenes	*GATA6*	28.6%		^50^
			*TWIST1*	50%		^50^

MF, mycosis fungoides; SS, Sézary syndrome; NA, data not available. *Methylation frequency refers to the proportion of cases with abnormal methylation of a specific gene in the CTCL cohorts, according to the references.

### DNA hypermethylation

In 2000, promoter hypermethylation-driven silencing of *CDKN2A* was reported in CTCL and was associated with aggressive disease^36^. The *CDKN2A* gene encodes a nuclear protein that can block cell cycle progression by effectively inhibiting the kinase activity of cyclin-dependent kinase 4/6, thereby exerting tumor suppressor functions. Subsequently, a large body of knowledge has gradually accumulated concerning the specific pattern of DNA hypermethylation in CTCL. The evidence indicates the epigenetic silencing of diverse tumor suppressor genes including those involved in cell cycle regulation (*CDKN2A, CDKN2B, CDKN1A,* and* RARB*), the insulin growth factor pathway (*IGF2* and* SOCS1*), pluripotent regulation of stem cells (*NEUROG1*), apoptosis signaling (*TP73, FAS,* and *TMS1*), cell differentiation (*PPARG*), DNA repair (*MGMT* and* MLH1*), chromosomal instability (*CHFR*), and other putative tumor suppressors (*BCL7A, THBS4, PTPRG, CMTM2, SHP-1, SAMHD1,* and *CD26*)^14,37–39^.

*MLH1* is a DNA mismatch repair gene that is involved in correcting mutations during DNA replication^40^. Promoter hypermethylation-mediated silencing of the *MLH1* gene was identified in 64% of CTCL patients showing microsatellite instability, suggesting that aberrant methylation of the *MLH1* gene promoter may be important in disease progression in a subset of CTCL patients^40^. The tumor suppressor, Src homology region 2 domain-containing phosphatase 1 (SHP-1), is an important negative regulator of cell signaling for the interleukin (IL)-2-mediated Janus kinase (JAK)/signal transducer and activator of transcription (STAT3) pathway^41^. Promoter hypermethylation-induced loss of SHP-1 has been frequently reported in the tumor stages of MF and MF cell lines^41,42^. This was associated with phosphorylated (p)-STAT3 activation, which induces DNMT1 to methylate the *SHP-1* promoter^41–44^. These results indicate that epigenetic silencing of SHP-1 may play a role in the pathogenesis of CTCLs by permitting the persistence of oncogenic STAT3 signaling and, possibly, other receptor complexes.

Resistance to apoptosis is a crucial mechanism for the accumulation of malignant T-cells in CTCL lesions. Deficiencies in FAS-mediated activation-induced cell death (AICD) play pivotal roles in CTCL pathogenesis^45^. Decreased or absent FAS expression, due to hypermethylation in five specific CpG dinucleotides in the *FAS* promoter, has been reported in a significant proportion of CTCL patients^17^. In a similar vein, inactivation of the *TP73* gene *via* promoter hypermethylation also contributes to the AICD resistance, facilitating the development of CTCL^14,46^.

The methylation of *PPARG*, a critical gene involved in cell differentiation, shows remarkable differences among stage I classical MFs with a distinct prognosis^37^. *PPARG* was demonstrated as a significant predictor of disease progression within the 6-year follow-up period, even after adjustment for patients’ age and sex^37^. A more recent study has shown that recurrent promoter hypermethylation of the chemokine-like factor, *CMTM2,* is sufficient to distinguish 15 SS patients from 7 erythrodermic inflammatory dermatosis patients with 100% specificity and 100% sensitivity, indicating its utility as an epigenetic clinical diagnostic marker for CTCL^47^.

### DNA hypomethylation

In contrast to promoter hypermethylation of specific tumor suppressors, aberrant transcriptional activation associated with DNA hypomethylation at specific loci in the promoters were reported in genes related to CTCL oncogenesis.

Ectopic plastin-3 (PLS3) expression was previously reported in Sézary cells and served as a biomarker for SS^48^. Jones et al.^49^ reported that the hypomethylation of CpG dinucleotides in the* PLS3* promoter induced abnormal activation of this non-lymphoid gene in CTCL cells. Accordingly, exposing PLS negative lymphoid cell lines to the hypomethylating agent, azacitidine, resulted in increased transcriptional activity of the *PLS3* promoter and upregulation of PLS3 expression^50^. Direct epigenetic modifications related to overexpression were identified in two other candidate oncogenes, *GATA6* and *TWIST1*^50^. Hypomethylation-mediated GATA6 activation induced aberrant CD137 ligand expression and promoted multiple tumor-driving pathways in the proliferation of CTCL cell lines and tumor formation in NSG and C57BL/6 mouse models^51^. Special AT-rich region binding protein 1 (SATB1), a global transcription regulator overexpressed in a portion of CD30+LPDs, was demonstrated to promote the proliferation of malignant CD30+ T cells^52^. Its upregulation has been associated with DNA demethylation on a specific CpG-rich region of the *SATB1* promoter^52,53^.

### DNA hydroxy-methylation

In 2009, two seminal studies describing the presence of 5-hydroxymethylcytosine (5-hmC) offered the first insights into the metabolism of 5-mC^54,55^. Ten eleven translocation (TET) 1-3 enzymes play a critical role in epigenetic stability by oxidizing 5-mC to 5-hmC^56^. Global loss of 5-hmC is an epigenetic biomarker in CTCL, including MF and CD30+LPDs^57,58^. The level of 5-hmC decreased along with disease progression in MF and was associated with poor prognosis^57^. NGS has identified somatic mutations in the *TET2* gene in a small subset of SS patients^59^. However, in MF and CD30+LPDs, the 5-hmC loss seemed to be in an TET-independent manner^57^. Thus, the relationship between *TET2* mutation and the loss of 5-hmC remains to be elucidated.

## Histone modification

Histone modifications have been related to many cellular processes during normal physiological growth and cancer. Chromatin has a dynamic configuration used to package DNA and organize the eukaryotic genome^60^. Its basic functional unit, the nucleosome, consists of a 147-base-pair segment of DNA wrapped around the octameric histone complex^61^. The histone octamer contains two copies of each histone (H2A, H2B, H3, and H4). Each possesses a globular central domain and a tail enriched in lysine and arginine residues. The N-terminal “tail” region projects from the nucleosome, which is accessible to post-translational modifications at specific amino acid residues^62^. More recent studies have revealed that the globular domains of histone can also be modified in the same way^63^. The considerable diversity in histone modifications introduces a remarkable complexity that is beginning to be elucidated. These modifications include acetylation, methylation, phosphorylation, ubiquitination, and sumoylation as well as uncommon variants, such as succinylation, butyrylation, and neddylation^64,65^. These modifications can influence gene expression either directly by modifying the histone-DNA interaction, or indirectly by altering recognition sites and accessibility for specific binding proteins, and further regulating gene expressions involved in critical cellular processes^66,67^. Notably, these modifications may also occur in nonhistone proteins to support their biological functions, such as transcription factors or cytoplastic proteins^68^. With the advent of chromatin immunoprecipitation coupled with DNA microarray analysis or massive parallel sequencing (ChIp-chip or ChIp-seq), the global profile of histone modifications in human cancers can be monitored^69^. Dysregulation in the pattern of histone modifications has been extensively correlated with neoplastic transformation and differs according to tumor type^66^. Acetylation and methylation, as two main types of histone modifications associated with oncogenesis, have been well-studied in CTCL.

### Histone acetylation

Acetylation of lysine on histone tails is highly dynamic and is important for the regulation of chromatin structure, gene transcription, and DNA repair^70^. Histone acetylation is a reversible process catalyzed by two opposing classes of enzymes, histone acetylases (HATs) and histone deacetylases (HDACs)^70^. HATs transfer acetyl groups from acetyl-CoA to the ε-amino group of lysine residues, which neutralizes the lysine positive charge and further untightens compact chromatin and enhances the accessibility of the transcriptional mechanism^67^. Conversely, HDACs catalyze the removal of the acetyl group from histones, leading to chromatin condensation and gene transcriptional repression^71,72^. The dynamic equilibrium of the two enzyme families plays a critical role in governing numerous cellular processes and disease states^73^. Dysregulated patterns of histone acetylation have been detected in a variety of cancers with gain-of-function mutations or overexpression of HDACs^74^. In recent decades, a great deal of attention has focused on interventions for aberrant enzymes to achieve normalcy. The use of HDACis leads to the accumulation of acetylated histones and the reverse of dysregulated expression of critical genes enriched in cellular processes such as apoptosis or cell cycle, as well as oncogenic signaling, such as mitogen-activated protein kinase/extracellular signal-regulated kinase (MAPK/ERK) and JAK/STAT pathways in CTCL^75^. HDACis have emerged as novel cancer therapeutic agents, especially for hematological malignancies that include CTCL (see HDACis section).

### Histone methylation

In contrast to histone acetylation, histone methylation changes the compaction status of the chromatin and creates docking sites in the chromatin that can be recognized by various proteins, such as transcription initiation factors^64^. The methyl groups are added to lysine and arginine residues in the histone tails^76^. Lysines may be mono-, di-, or tri-methylated, and arginine residues may be symmetrically or asymmetrically methylated^77^. This varied methylation pattern leads to the activation or repression of gene expression, depending on the residue that is methylated^77^. Histone methylation regulates many biological functions that are crucial for normal cell differentiation, and has a central role in carcinogenesis and tumor progression^77,78^. Lysine-specific histone demethylase 1A (LSD1) is the first reported histone demethylase that can induce gene silencing as a subunit of the transcriptional corepressor “coREST”^79^. Domatinostat, a novel HDACi that also targets LSD1, induces cell cycle arrest in the G2/M phase and decreases the growth of CTCL cells^80^. Polycomb group proteins are the most studied histone-associated proteins in cancer. Enhancer of zeste homolog 2 (EZH2), the catalytic component of polycomb repressive complex 2, contains a catalytic SET domain that mediates histone H3 lysine 27 trimethylation to induce transcriptional silencing^81^. Heterozygous missense mutations resulting in the substitution of tyrosine 641 (Y641) within the SET domain of EZH2 were noted in 22% of patients with diffuse large B-cell lymphoma (DLBCL), and this mutation conferred increased histone methylation catalytic activity, while loss-of-function mutations in EZH2 conferred a poor prognosis in myeloid malignancies and T-cell acute lymphoblastic leukemia^82–84^. EZH2 inhibitors, including tazemetostat, valemetostat, CPI-1205, and GSK2816126, induce proliferation arrest, differentiation, and eventual apoptosis of DLBCL cells over the course of several days, and have shown encouraging preliminary results in clinical trials^85–88^. However, NGS studies in CTCL failed to find recurrent mutations in the *EZH2* gene. Our group identified overexpression of EZH2 in the CD30+ anaplastic T cells in PCALCLs, and showed that targeting EZH2 catalytic activity exerts a direct antitumor cell effect and also promotes T-cell-mediated immunity at the tumor site, which has significant implications for treating cutaneous CD30+LPDs as well as transformed MF^75^.

## MiRNAs

MiRNAs are small, single-stranded, non-coding RNAs that are 18 to 25 nucleotides in length. They have a broad impact on gene regulation at post-transcriptional levels^89,90^. There are approximately 1,000 miRNA genes in the human genome with evolutionary conservation^91^. DNA encoding these miRNAs are located in the exons or introns of protein-coding genes (70%), encoding host messenger RNAs (mRNAs) and miRNAs simultaneously, or within intergenic areas (30%) as independent transcription units for miRNAs^92^. Mature miRNA products recognize specific sequences in the 3′ untranslated regions of their target mRNAs and repress gene expression by blocking mRNA translation or inducing mRNA degradation^89^. Interestingly, each miRNA has the potential to modulate more than one target gene, and multiple miRNAs can regulate the expression of a single target mRNA, illustrating a robust network of miRNA regulation^91^. MiRNAs have broad roles in various biological processes, including apoptosis, differentiation, proliferation, and metabolism. They have been increasingly recognized as being vital for normal development and may be compromised in diseases such as cancer^93^. Evidence has demonstrated dysregulation of miRNAs in a broad range of cancers including CTCL^94,95^. MiRNAs can be upregulated or downregulated in tumor tissues, although a greater proportion of miRNAs seems to be overexpressed, rather than underexpressed, in cancer^94^. Recent large-scale sequencing studies have gradually elucidated the global miRNA landscape in CTCL. In **Table 2**, we list upregulated or downregulated miRNAs found in CTCL patients or CTCL cell lines, and review their functions as either oncogenic miRNAs, tumor-suppressive miRNAs, or diagnostic/prognostic markers, according to their impact on respective target genes.

**Table 2:** The functional microRNA aberrations in cutaneous T-cell lymphomas

MicroRNA	Expression	Function*	Confirmed target*	Potential clinical value	Disease	References
MiR-155	Over-	OG	*SATB1*	Phase 1 trial of cobomarsen	MFt, SS, PCALCL	^97–101^
MiR-106b	Over-	OG	*PTEN*		SS	^174^
MiR-199a-3p	Over-	Unidentified	*EVL*		SS	^105,175^
MiR-181a/b	Over-	Unidentified	*SAMHD1*		MFt, SS	^97,175,176^
MiR-125b-5p		OG	*MAD4*	Chemotherapy resistance	CTCL	^110^
MiR-122	Over-	OG		Chemotherapy resistance	MFt	^109^
MiR-21	Over-	OG		Prognosis	MFt, SS, PCALCL	^97,104,105^
MiR-486	Over-	OG			SS	^104^
MiR-142-3p/5p	Over-	OG			MFt, PCALCL	^97,104^
MiR-214	Over-	OG			SS	^104,106^
MiR-150	Down-	TS	*CCR6*		Stage IV MF, SS	^114,175^
MiR-29b	Down-	TS	*BRD4*		MF, SS, PCALCL	^15,111^
MiR-16	Down-	TS	*BMI1*		MF, SS	^117,175^
MiR-223	Down-	TS	*TOX*		MF, SS	^112,175^
MiR-200c	Down-	TS	*JAG1*		MF, SS, PCALCL	^97,116^
Let-7a, let-7d and let-7f	Down-	Unidentified		Let-7a for prognosis	MFt	^127^

OG, oncogene; MFt, tumor-stage mycosis fungoides; SS, Sézary syndrome; PCALCL, primary cutaneous anaplastic large cell lymphoma; CTCL, cutaneous T-cell lymphoma; MF, mycosis fungoides; TS, tumor suppressor. *All of the confirmed targets and functions of microRNAs have been validated by experimental data of CTCLs.

### Oncogenic miRNAs

A portion of miRNAs can act as oncogenic miRNAs (onco-miRNAs). Their overexpression inhibits the expression of tumor suppressor genes and contributes to the initiation or development of cancers. The list of onco-miRNAs in CTCL is short (**Table 2**), but their broad impact should be emphasized with a potential role in the clinical setting.

MiR-155 is one of the first described onco-miRNAs in the context of cancers^96^. Aberrant overexpression of miR-155 has been described in multiple cohorts of CTCL patients^97–101^. Several studies demonstrated increased miR-155 levels from skin-limited CTCL to advanced stages, suggesting that miR-155 is involved in tumor progression of MF^98^. The upregulation of miR-155 was induced by increased transcription of the miR-155 precursor gene, *BIC* (B-cell integration cluster) and highly activated transcription factor STAT5 in malignant T cells^102^. STAT5-mediated miR-155 expression directly represses the promoter region of the putative tumor suppressor *SATB1*, which in turn enhanced the expression of cytokines characteristic of T helper 2 (Th2) polarization, including IL-5 and IL-9^101^. The oncogenic role of miR-155 was investigated by transducing anti-miR-155 into MyLa cells^103^. Increased G2/M cell cycle arrest and increased apoptosis were observed in response to vorinostat and SL111 (an inducer of cell cycle arrest) treatment, as well as reduced tumor formation in xenograft models after blocking miR-155^103^. Transcriptome profiling before and after treatment with cobomarsen (an optimized anti-miR-155) identified miR-155 downstream targets involved in multiple survival pathways associated with CTCL oncogenesis, including JAK/STAT, MAPK/ERK, and phosphatidylinositol 3-kinase (PI3K)/protein kinase B (AKT) pathways^99^. The upregulation of miR-21 was identified in Sézary cells and was associated with poor outcomes in a subset of SS patients^104^. Another study demonstrated that constitutively activated STAT3 directly bound to the promoter of the precursor gene of miR-21, resulting in the upregulation of miR-21 and induction of malignant T-cell survival and apoptotic resistance of Sézary cells^105^. Deep-sequencing analysis identified increased miR-214 expressions in purified CD4+ T cells from SS patients, compared with that in patients with erythroderma dermatitis and healthy controls^106^. Further studies demonstrated that miR-214 was upregulated *via* transcription factor twist-related protein 1 (TWIST1), incorporating the tumor-promoting factor bromodomain-containing protein 4 (BRD4)^107^. Both miR-214 antagonist and BRD4 inhibitor decreased the tumor severity in CTCL mouse models^107^.

In addition to its oncogenic role, several miRNA signatures are involved in chemotherapy resistance, which remains a severe barrier to efficient antitumor therapies^108^. In CTCL, upregulated miR-122 was shown to block chemotherapy-induced apoptosis by enhancing the anti-apoptotic AKT/P53 pathway in CTCL cells^109^. Bortezomib treatment suppressed Myc proto-oncogene protein (MYC) that, in turn, transcriptionally induced miR-125b-5p to block drug-induced apoptosis, thereby reducing the drug sensitivity of CTCL cells to bortezomib^110^.

### Tumor-suppressive miRNAs

Several miRNAs exhibit tumor-suppressive effects in CTCL. Their generally weak or absent expression in many subsets of CTCL patients have been closely correlated with the malignant transformation of T cells.

Decreased expression of miR-29b was found in the miRNome profile of CTCL patients compared to the profile of healthy controls^15,111^. Diminished miR-29b reportedly induced the accumulation of tumor-promoting protein BRD4, which bound acetylated histones throughout the genome to regulate other tumor-related genes and highly activate IL-15 signaling, which, in turn, led to aberrant expression of miR-29b^15^. Bortezomib was shown to increase miR-29b expression and block the IL-15/miR-29b/BDR4 loop *in vitro* and prevent tumor progression in murine models of CTCL^15^. MiR-233 expression was downregulated in both skin lesions and peripheral blood mononuclear cells from a proportion of MF patients and further decreased as the clinical stage advanced^112^. Diminished miR-233 was correlated with the overexpression of the oncogenic transcription factor, E2F1, and the myocyte-specific enhancer factor, 2C (MEF2C)^112^. These authors and others reported that miR-233 also negatively targets *TOX*, which encodes an essential transcription factor in CD4+ T cell development and plays a critical role in the pathogenesis of MF with aberrantly increased expression in early MF^112,113^. MiR-150 was identified as a tumor-suppressive miRNA in advanced CTCL, as significant downregulation was found in purified malignant cells from advanced MF and SS patients, compared with normal T cells from healthy controls^114^. The authors also reported that miR-150 directly targets *CCR6*^114^. The repressed expression of miR-150 upregulated C-C motif chemokine 20 (CCL20)-C-C chemokine receptor type 6 (CCR6) interaction and inhibited CTCL cell migration *in vitro* and* in vivo*^114^. Furthermore, the pan-HDACis, vorinostat and panobinostat, inhibited metastasis by restoring miR-150 in advanced CTCL^115^. Hypermethylated silencing of miR-200c in some MF patients was associated with overexpression of the Notch-ligand protein jagged-1 (JAG1) and activation of neurogenic locus notch homolog protein 1 (NOTCH1) in MF^116^. The lack of miR-16 expression in CTCL was implicated in overcoming cellular senescence, which is an early step in carcinogenesis^117^. Forced expression of miR-16 enhanced p21 expression by downregulation of the polycomb group protein BMI-1, thereby inducing cellular senescence^117^. Intriguingly, this process was recapitulated by vorinostat treatment in CTCL cells^117^.

### MiRNAs as diagnostic/prognostic markers

The potential of miRNAs as biomarkers is based on their small size, relatively limited numbers, and stability in a variety of biological specimens that include tissue, blood, and stool^118,119^. A number of studies have tried to evaluate the potential of miRNAs as diagnostic or prognostic markers in CTCL. However, only a few studies have involved large patient cohorts, precisely defined patient populations, and independent validation cohorts^120–122^. So far, none of these biomarkers has met the key requirements for adoption in the clinical setting.

In disease diagnosis, various combinations of 3 to 5 miRNAs, the so-called “miRNA classifiers”, were investigated as novel diagnostic markers. Ralfkiaer et al.^120^ reported that upregulated miR-155 and 2 dysregulated miRNAs (miR-203 and miR-205) could distinguish early MF patients with subtle histopathological changes from benign inflammatory disease donors with a sensitivity of 91% and specificity of 97% in their cohort. This result was validated in another cohort of 30 CTCL patients with similar desirable results^123^. Dusílková et al.^124^ established a diagnostic plasma miRNA classifier based on 5 miRNAs by adding reductions of miR-22/miR-223 to the miR-155/miR-203/miR-205 classifier. The authors reported increased sensitivity (94%) and specificity (100%) in a small cohort^124^. In a more recent study involving 154 CTCL patients, a set of 5 miRNAs (miR-200b, miR-203, miR130b, miR-142-3p, and miR-155) successfully classified CTCL subtypes including early stage MF, advanced stage MF, and other subtypes of CTCL with a sensitivity of 93%, 96%, and 97.5%, respectively, and a specificity of 80%^121^. Erythrodermic MF and SS are difficult to clinically distinguish because of their similar clinical and histological features^125^. Rittig et al.^126^ retrospectively compared the miRNA expression profiles between these two subtypes and found 27 differentially expressed miRNAs. Further screening of miRNA collections as diagnostic classifiers requires validation in prospective cohorts. A group of miRNAs (miR-29b, miR-155, miR-27b, miR-93, and miR-92a) showed differential expression between PCALCL lesions and tumor-stage MF in a small proportion of patients^111^. Whether these miRNAs can serve as diagnostic markers to classify PCALCL and MF remains to be determined in studies with larger cohorts.

The prognostic value of miRNA signatures has been extensively investigated in the past decade. MiR-21 upregulation has been associated with unfavorable outcomes in MF patients^104^. Maj et al.^127^ found that let-7a was downregulated in advanced stages of MF. The level of let-7a expression was an independent unfavorable prognostic indicator, based on data of univariate survival analysis and a multivariate Cox regression model^127^. MiR-155 and miR-200b were associated with 5-year overall survival (OS) in a CTCL cohort, outperforming the Ki-67 prognostic mediator^121^. When combined with Ki-67, miRNA expression generated a prognostic classifier to predict 5-year OS with 77% sensitivity and 81.1% specificity^121^. A classifier based on 3 miRNAs (miR-106b-5p, miR-148a-3p, and miR338-3p) distinguished the high risk group in a cohort of 154 patients with early stage MF^122^.

## Chromatin-remodeling complex

DNA is organized as chromatin, which maintains the dynamic balance between the compact structure and open state^60^. Alteration of chromatin, as an early but complex step in the control of genome-wide gene expression, requires multiple regulatory elements, including histone modifications and chromatin-remodeling complexes (i.e., remodelers)^64,128^. Remodelers utilize ATP-hydrolytic energy to move, eject, and restructure the packed or unpacked DNA in nucleosomes. The remodelers participate in DNA-template processes, including replication, transcription, and repair^128^. Concerning transcriptional regulation, remodelers can physically interact with other transcription factors to induce transcriptional activation or repression. These interactions are crucial in multiple cancers, including CTCL^12^. Remodelers can be classified into 4 distinct families based on their compositions: switching defective/sucrose nonfermenting (SWI/SNF), imitation switch (ISWI), chromodomain-helicase DNA-binding protein (CHD), and inositol requiring 80 (INO80)^128^. The SWI/SNF complex is one of the most well-studied human remodelers characteristic of tumor suppressive effects in multiple cancer types^129^. SNF5 is one of the main central subunits of the SWI/SNF complex and is mutated in malignancies^129^. SNF5 deficiency mediates SATB1 downregulation *via* physical binding and induces apoptosis resistance in Sézary cells^101^. SATB1 is a nuclear matrix-associated protein that acts as a T-cell lineage-specific chromatin organizer^130^. SATB1 provides a docking platform for a variety of chromatin-remodeling factors, folds chromatin into loops, and binds to specific DNA elements, thereby regulating the transcription of various genes associated with T-cell development and differentiation^130^. Aberrant SATB1 expression has been identified in multiple subtypes of CTCLs, with context-dependent functions. The loss of SATB1 expression was first identified in malignant SS cells versus normal CD4+ T cells. The expression was functionally correlated with resistance to apoptosis through the regulation of *FASL/CD95L* transcription^131^. Multiple groups have identified the attenuation of SATB1 in MF and its relationship to disease progression and Th2 polarization, supporting the tumor-suppressive function of SATB1 in CTCLs^101,132,133^. In contrast, SATB1 overexpression associated with promoter demethylation was demonstrated in the CD30+ anaplastic T cells in a portion of CD30+LPDs, and was shown to promote CD30+ cell proliferation by inhibiting G1 cell cycle arrest, suggesting a complicated and context-dependent role of SATB1 in malignant T cells^52,75^. The role of chromatin remodelers in CTCL remains unclear and further in-depth investigations are required.

## Targeting epigenetic modifications in CTCL treatments

### DNMT inhibitors

The major tumor-specific DNA methylation profile of CTCL features the significant hypermethylation of tumor suppressor genes. This profile information has facilitated the development of novel therapeutic strategies. DNMT inhibitors that induce hypomethylation, azacitidine, and decitabine, represent the first class of epigenetic-modulating drugs to be approved by the FDA for hematological malignancies^18,134^. Abundant research data have demonstrated that DNA demethylating agents can de-repress methylation-silenced genes in CTCL cell lines^14,37^. Preclinical studies have demonstrated the promising efficacy of these drugs in advanced CTCL^135,136^. An ongoing phase I trial of decitabine plus pembrolizumab for CTCL and PTCL are of interest (NCT03240211).

In addition, methotrexate (MTX), an antimetabolite that blocks the action of dihydrofolate reductase and is widely used in treating CTCL, was recently proposed as an epigenetic regulator capable of blocking the synthesis of methionine, thereby reducing the level of S-adenosylmethionine, the major methyl group donor of DNMTs^137^. MTX disturbs the methylation of the *FAS* promoter in malignant lymphocytes^38^. MTX and its more potent analog pralatrexate may be valuable as novel agents to correct aberrant DNA methylation in CTCL^137^.

### HDACis

Vorinostat, a pan-HDACi, was the first to be approved by the FDA in 2006 for the treatment of R/R CTCL patients^20^. The selective HDACi, romidepsin, which targets HDAC1 and 2, was approved for CTCL and peripheral T-cell lymphoma (PTCL) patients in 2009 (**Table 3**)^77,138^. Vorinostat and romidepsin have achieved clinical efficacies of 24.2% to 34.0% in terms of overall response rate (ORR). Both drugs are well tolerated with mild to modest side effects, including thrombocytopenia, fatigue, and gastrointestinal disturbance^139–141^. Belinostat was approved for R/R PTCL and CTCL in 2015, based on its promising efficacy and manageable safety profile^142,143^. More recently, chidamide, a novel benzamide class of selective HDACi that inhibits HDAC1, HDAC2, HDAC3, and HDAC10, was approved by the National Medical Products Administration (NMPA) (China) for the treatment of R/R PTCL and CTCL^144^. A 28% ORR and 14% complete remission rate were reported for chidamide^144^.

**Table 3:** Histone deacetylase inhibitors approved or in ongoing clinical trials in cutaneous T-cell lymphomas

Agent	Specificity	Structural class	Route	Clinical status in CTCL	References
Vorinostat	Pan	Hydroxamic acid	Oral	FDA-approved	^140^
Romidepsin	HDAC1/2	Bicyclic peptide	IV	FDA-approved	^21^
Belinostat	Pan	Hydroxamic acid	IV	FDA-approved	^142^
Chidamide	HDAC1/2/3/10	Benzamides	Oral	NMPA-approved	^144^
Panobinostat	Pan	Hydroxamic acid	Oral	Phase II trial—R/R CTCL	^145^
SHP-141	Unknown	Hydroxamic acid	Topical	Phase II trial—stage IA-IB CTCL	^147^

CTCL, cutaneous T-cell lymphoma; FDA, Food and Drug Administration; HDAC, histone deacetylase; IV, intravenous; NMPA, National Medical Products Administration (China); R/R, refractory/recurrent.

Other promising HDACis are being evaluated in ongoing clinical trials for advanced CTCL (**Table 3**). In addition to its established role in multiple myeloma, panobinostat displayed an ORR of 17.3% in a phase II trial for R/R CTCL patients^145,146^. SHP-141 gel, an HDACi agent used for the topical treatment of early stage skin-limited CTCL, has notably also demonstrated a skin-restricted response in stage IA–IB CTCL patients with no systemic toxicity in phase I and II trials^147,148^.

New multi-targeting HDACis against multiple biological targets has been developed in recent years. A dual HDAC/PI3K inhibitor, CUDC-907, was evaluated in a phase I clinical trial for R/R lymphoma and multiple myeloma^149^. It showed some efficacy in patients with DLBCL^149^. However, only 2 T-cell lymphoma patients were recruited, and they showed little response to this compound^149^.

In addition, combination therapies of HDACi and conventional therapy have been widely investigated in CTCL. It is likely that many epigenetic drugs offer synergistic benefits with conventional chemotherapies. This strategy of combination therapy may increase therapeutic efficacy and reduce the likelihood of drug resistance. Several case reports have proposed stronger therapeutic benefits with good tolerance when using HDACis combined with other systemic treatments such as interferon-gamma^150,151^. Vorinostat combined with bexarotene for refractory CTCL has been identified as the maximum tolerated dose in a phase I trial, and clinical efficacy of this combination therapy is anticipated^152^. A recent phase I study on R/R CTCL subjects showed promising clinical safety and efficacy of the combined regimen of romidepsin plus liposomal doxorubicin^153^. These studies may facilitate future combination therapies, demonstrating the added benefit of epigenetic agents and conventional medicines.

### Targeting miRNAs

The miRNAs have demonstrated important roles in CTCL pathogenesis and disease progression thus promoting the idea that they are potentially attractive therapeutic targets. Two treatment approaches are currently used in miRNA-targeted therapies in CTCLs. In one approach, the upregulation of onco-miRNAs is inhibited by an oligonucleotide antagonist. In the other approach, the dysregulated miRNA expression is reversed by existing drugs. Cobomarsen, an oligonucleotide inhibitor of miR-155, was shown to regulate multiple survival pathways and reduce the proliferation and survival of CTCL cells^99^. Good tolerance and clinical activity of cobomarsen were reported in preliminary data from a phase II trial for patients with MF stages I–III (NCT02580552). The trial is ongoing and the final safety and efficacy data are anticipated^154^. Other than the antisense oligonucleotides, reversal of miR-214 by a BRD4 inhibitor, restoration of miR-29b by bortezomib, as well as upregulation of miR-16 and miR-150 by vorinostat have been reported^15,107,115,117^. The findings highlight the potential of miRNA-targeted treatment as a novel supplement to improve the therapeutic sensitivity of current antitumor agents for R/R CTCL.

## Perspectives and conclusions

### Crosstalk between epigenetic regulators and their therapeutic potentials

In the past decade, an improved understanding of epigenetic regulatory mechanisms, particularly cancer-specific epigenetic alterations, has radically altered views concerning the understanding and treatment of CTCL. Epigenetic alterations in DNA methylation, histone modification, miRNAs, and chromatin-remodeling complexes, are involved in the regulation of apoptotic resistance, cell cycle arrest, and triggering cellular pathways related to the development of CTCL. These events facilitate the neoplastic transformation in CTCL. Notably, crosstalk within the epigenetic network has been demonstrated in both normal and malignant cells^155^. These epigenetic modifiers function in an orchestrated manner to fine-tune a complex regulatory network with important crosstalk between them. For example, DNA methylation status is an epigenetic checkpoint that silences the expression of tumor suppressor miR-220c in MF tumor-stage samples and CTCL cell lines^116^. MiR-155 deficiency reportedly increases the sensitivity to vorinostat in CTCL cells, with reversal of aberrant miR-16 expression by vorinostat *in vitro*^103,117^. These interactions add another layer of complexity to the epigenetic regulation of CTCL. This raises the more fundamental issue of rationally designed combination epigenetic therapies, because the efficacy of epigenetic-based monotherapies in CTCL is limited^139,140^. Emerging therapeutic strategies that take advantage of crosstalk between different epigenetic mechanisms have been developed, including multi-compound drugs^156^. Preclinical studies revealed synergistic epigenetic modulatory effects of romidepsin and azacitidine on CTCL^157^. Hydralazine/valproate (TRANSKRIP™), a multi-compound agent packaging an HDACi valproate together with the DNMT inhibitor hydralazine, was well tolerated and efficient, with an ORR of 71% reported in a phase II study for R/R CTCL^158^. Clinical trials regarding the combined application of “epi-drugs” in CTCL are expected.

### Discovering predictive biomarkers for epi-drug responses

In part due to the heterogeneity of the genome/transcriptome/epigenome of individual patients, the response rates of current single agent epigenetic drugs are relatively low. Precise predictive indicators for therapeutic responses are a recognized unmet need^140^. Downregulated expression of the *BCL11B* gene^159^, overexpression of *LAIR2*^160^, or recurrent genetic alterations (*RAD23B* copy number loss and *STAT3* Y640F variant^161^) have been reported as potential indicators of HDACi resistance in laboratory investigations. Using the assay for transposase-accessible chromatin sequencing (ATAC-seq) that can map the open chromatin sites throughout the genome, a seminal study indicated that only patients who responded well to HDACis showed a gain of chromatin accessibility in CTCL cells after treatment^162,163^. The utilization of multiple NGS technologies is required to reveal the multi-omics features that are interfered by epigenetic drugs. Drug sensitivity tests and drug screenings based on the individual genome/epigenome maps in larger cohorts are needed to identify unified and precise predictive biomarkers to assist drug selection in advanced CTCL patients.

### Tumor microenvironment and epigenetic modifiers

Epigenetic plasticity interferes with the progress of cancer cell development and also reengineers the tumor microenvironment^164,165^. Immune cells that infiltrate around malignant T cells in the skin are also vulnerable to epigenetic modifiers. A recent study showed that HDACi-induced chromatin accessibility was greater in host T cells than in CTCL cells, suggesting an essential role of host immune cells in epigenetic therapy^162^. EZH2 and DNMT inhibitors play an essential role in enhancing host immunity against cancer in preclinical models^166,167^. Our recent study showed that EZH2 inhibitors derepressed C-X-C motif chemokine ligand 10 (CXCL10) and facilitated the recruitment of effector CD4+ and CD8+ T cells into the tumor microenvironment *via* a CXCL10/C-X-C motif chemokine receptor 3 (CXCR3) interaction in PCALCL^75^. However, the complex interplay between malignant T cells and tumor-infiltrating immune cells in CTCL and their implications for epigenetic modifications remains largely unknown and merits exploration in the future.

### Discovering new epigenetic modifications in CTCL

Several epigenetic pathways with prominent biological functions have emerged and are of great interest in the field of oncology. These include long non-coding RNA (lncRNA), N6-methyladenine (m6A), and 5-methylcytosine (m5C) modification of RNA^168–170^. Transcriptome sequencing has revealed the dysregulation of several lncRNAs in CTCL^171^. However, their functions and clinical implications remain to be elucidated^171^. Exploring these newly defined epigenetic pathways in CTCL may help to replenish their specific epigenome spectrum and shed light on the molecular pathogenesis of this disease.

In conclusion, we are entering a very exciting era of epigenetics in CTCL, although our understanding of epigenome alterations in CTCL is at the very early stages. Improved understanding of epigenetic plasticity could advance diagnostic strategies and yield new therapeutic regimens that exploit the vulnerabilities of transformed malignant T cells.
